# Changes in the prevalence of hyperuricemia in clients of health examination in Eastern China, 2009 to 2019

**DOI:** 10.1186/s12902-022-01118-z

**Published:** 2022-08-10

**Authors:** Dunmin She, Yongliang Wang, Jing Liu, Na Luo, Shangyong Feng, Ying Li, Jin Xu, Shichun Xie, Yan Zhu, Ying Xue, Zhenwen Zhang

**Affiliations:** 1 Clinical Medical College, Yangzhou University, Yangzhou, 225001 Jiangsu China; 2 Department of Endocrinology, Northern Jiangsu People’s Hospital Affiliated to Yangzhou University, Yangzhou, 225001 Jiangsu China; 3 Dalian Medical University, Dalian, 116000 Liaoning China; 4 Department of Information Center, Northern Jiangsu People’s Hospital Affiliated to Yangzhou University, Yangzhou, 225001 Jiangsu China; 5 Department of Endocrinology and Metabolism, Tongji Hospital, School of Medicine, Tongji University, Shanghai, 200065 China

**Keywords:** Prevalence, Hyperuricemia, Serum uric acid level, Age, Body mass index

## Abstract

**Background:**

With the continuous improvement of people's living standards, the incidence of hyperuricemia (HUA) is increasing globally. The prevalence of HUA ranged in terms of region, race, and age. This study aims to investigate the changes in the prevalence of HUA in clients of health examination in Eastern China between 2009 and 2019.

**Methods:**

Chinese men and women aged 20–79 years (*n* = 4847 in the 2009 group and *n* = 12,188 in 2019 group) who had received health examinations were enrolled. Serum uric acid (UA) levels and biochemical parameters, including fasting blood-glucose (FBG), triglyceride (TG), total cholesterol (CHOL), high-density lipoprotein (HDL), low-density lipoprotein (LDL), creatinine (Cr) and blood urea nitrogen (BUN) were evaluated. The prevalence of HUA in different age groups were measured, and the correlation of biochemical parameters with HUA were analyzed.

**Results:**

The prevalence of HUA was 18.7% in the 2019 group, which was significantly higher than that in 2009 (11.1%). In females, the prevalence of HUA was significantly higher in 2019 than 2009 for age groups of 20–29 and 30–39 years. In male population, 2019 participants had significantly higher age-specific prevalence for all age groups than 2009 participants. Young men aged 20–29 years became the main population of HUA in the 2019 participants, whereas middle-aged men aged 40–49 years had the highest prevalence of HUA in the 2009 participants. The prevalence rates of HUA in all BMI groups in 2019 participants were significantly higher than those in 2009 participants. Spearmen’s correlation analysis and Logistic regression analysis indicated that BMI was positively correlated with HUA. The receiver-operating characteristic curve (ROC) analysis showed BMI > 24.48 kg/m2 and BMI > 23.84 kg/m2 displayed good capacities to discriminate the population with HUA from those without HUA in 2009 and 2019 participants, respectively.

**Conclusions:**

In recent 10 years, the prevalence of HUA was increased rapidly in Chinese adults, especially in males. In 2019, the young male group (20–29 years old) replaced the middle-aged male group (40–49 years old) in 2009 as the leading age group for male HUA. BMI was positively correlated with HUA, and might be a potential risk factors to predict HUA.

## Background

Uric acid (2,6,8 trioxypurine-C5H4N4O3, UA) is the final enzymatic product of purine metabolism in the body [1]. It can come from the human body or from the decomposition and metabolism of purines in food. Hyperuricemia (HUA) occurs due to increased UA production, impaired renal UA excretion, or a combination of both [2]. HUA is defined as blood uric acid concentration equals to and greater than 420 μmol/L in males and 360 μmol/L in females [3]. Elevated blood UA levels not only induce gouty arthritis, urolithiasis and UA nephropathy [4–6], but are also associated with type 2 diabetes, hypertension, cardiovascular disease (CVD) and chronic kidney disease (CKD) [7–10].

The prevalence of HUA ranged from 2.6% to 40% in terms of region, race, and research method [11]. The prevalence of HUA in some developed countries, such as USA (21.4%) and Japan (25.8%), is much higher than that of developing countries including Saudi Arabia (8.4%), Thailand (10.6%) and Turkey (12.1%) [12, 13]. Due to the vast territory of China, the prevalence of HUA varies significantly in different geographic regions. Generally, HUA is more common in cities than in rural regions, and higher in southern areas than in northern areas [14]. The prevalence of HUA in some economically developed regions of China, such as Guangdong (19.2%) [15], Beijing (17.9%) [16], and Hainan (25.1%) [17], is similar to that of developed countries [12, 13]. However, in some underdeveloped areas of China, the prevalence of HUA is much lower than those of developed regions in China during the same period [18, 19]. A previous study showed that the combined prevalence of HUA in rural areas was 11.7% in China [3]. A population-based study reported that the prevalence of HUA among the middle-aged and elderly people in Tibet was only 2.05% [20]. Although recent studies have described the prevalence of HUA in China [21–24], there is still a lack of research on the changing trend of the prevalence of HUA in China over the past 10 years, especially the characteristics of the prevalence of HUA among people of different sexes and ages. The aims of this study were to compare the prevalence of HUA in different age groups in the 2009 participants and 2019 participants, and to analyze the correlations between HUA biochemical parameters and HUA in Chinese adults.

## Methods

### Study population

Participants were Chinese men and women who had underwent health examinations in Northern Jiangsu People’s Hospital in 2009 and 2019 (*n* = 4847 in the 2009 group and *n* = 12,188 in 2019 group). The 2009 participants and the 2019 subjects are two completely different samples. The inclusion criteria were individuals aged 20–79 years and those who consented to participate in this study. The exclusion criteria were those who were under 18 years old and who were underweight (BMI < 18.5 kg/m2). All participants agreed to participate in the study and the study was approved by the Ethical Committee of Northern Jiangsu People’s Hospital.

### Data collection

The following data were collected: date of birth, sex, body height (BH, m), body weight (BW, kg), systolic blood pressure (SBP, mmHg), diastolic blood pressure (DBP, mmHg) and blood biochemical tests. BH, BW, and BP were measured according to standardized protocols. All blood tests were performed at the clinical laboratory of Northern Jiangsu Hospital. Fasting venous blood samples were obtained from physical examination participants and serum uric acid levels [UA reference range, 143–339 μmol/L (2.4–5.7 mg/dL)] were measured using an automated biochemical analyzer (Cobas 8000; Roche, Switzerland) [25]. Other blood biochemical parameters, including fasting blood glucose [FBG, 3.9–6.1 mmol/L ( 2.4–5.7 mg/dL)], triglyceride [TG, < 1.7 mmol/L (< 150.5 mg/dL), total cholesterol [CHOL, < 5.17 mmol/L (200 mg/dL)], high-density lipoprotein [HDL, 1.29–1.55 mmol/L (50–60 mg/dL), low-density lipoprotein [LDL, < 3.37 mmol/L (128 mg/dL)], creatinine [Cr, 44–133 μmol/L (0.5–15 mg/dL)], and blood urea nitrogen [BUN, 3.1–8.0 mmol/L (8.4–22.5 mg/dL)], were also determined using the same automatic biochemical analyzer (Cobas 8000; Roche, Switzerland).

### Definition and grouping

HUA was defined as serum UA ≥ 420.0 μmol/L (7.06 mg/dL) in males and ≥ 360 μmol/L (6.05 mg/dL) in female subjects [26]. Body mass index (BMI, kg/m^2^) was calculated using the following formula: BMI (kg/m^2^) = weight (kg) / height^2^ (m^2^). BMI was divided into normal weight (18.5 kg/m^2^ ≤ BMI < 24.0 kg/m^2^), overweight (24.0 kg/m^2^ ≤ BMI < 28.0 kg/m^2^), and obesity (BMI ≥ 28.0 kg/m^2^) according to the revised Asia–Pacific BMI criteria published by the World Health Organization (WHO) [27]. All the participants were divided into six age categories: 20–29 years, 30–39 years, 40–49 years, 50–59 years, 60–69 years and 70–79 years old.

### Statistical analysis

Statistical package for social sciences (SPSS) 25.0 was used for the statistical analysis in this study. All continuous variables were presented as mean ± standard deviation. Chi-square test was used to compare categorical variables, and independent-Samples T-Test was for continuous variables between two groups. Spearman correlations and binary logistic regression analysis were performed to explore the risk factors of HUA. Receiver-operating characteristic (ROC) curve analysis was used to determine the cut-off value, sensitivity and specificity of the risk factors which can potentially predict HUA. Tests were two-sided and a *p*-value < 0.05 was considered significant.

## Results

### Clinical characteristics of participants in the 2009 and 2019

Clinical characteristics of participants in the 2009 and 2019 participants were shown in Table 1. In the 2009 group, 1682 were females and 3165 were males, while 3852 females and 8336 males were included in the 2019 group. The participants in the 2009 were significantly older than those in the 2019 (46.6 ± 13.9 years vs. 45.3 ± 13.3 years, *p* < 0.01). BMI, levels of DBP, FBG, CHOL and BUN were significantly lower in the 2019 participants than those in the 2009, whereas levels of SBP, TG, HDL, LDL and Cr were significantly higher in 2019 participants than those in the 2009. In addition, serum UA level of participants in the 2019 was significantly higher than that in the 2009 [339.4 ± 85.4 μmol/L vs 314.5 ± 80.8 μmol/L (5.71 ± 1.44 mg/dL vs 5.27 ± 1.36 mg/dL), *p* < 0.01).

**Table 1:** Baseline characteristics of participants enrolled in 2009 and 2019

Variables	2009 participants			2019 participants		
	HUA (*n* = 538)	Non- HUA (*n* = 4309)	Total (*n* = 4847)	HUA (*n* = 2282)	Non- HUA (*n* = 9906)	Total (*n* = 12,188)
F/M	64/474	1618/2691^*^	1682/3165	214/2068	3638/6268^*^	3852/8336
Mean age	49.3 ± 13.9	46.3 ± 13.9^*^	46.6 ± 13.9	44.5 ± 13.6	45.5 ± 13.2^*^	45.3 ± 13.3^△^
BMI (kg/m2)	25.7 ± 2.7	23.4 ± 3.0^*^	23.9 ± 3.0	25.7 ± 3.1	23.7 ± 3.1 ^*^	23.6 ± 3.2^△^
SBP (mmHg)	128.5 ± 16.6	121.0 ± 22.9^*^	121.8 ± 22.4	136.3 ± + 17.1	131.0 ± 20.7^*^	132.0 ± 20.2^△^
DBP (mmHg)	86.5 ± 10.8	80.2 ± 10.8^*^	80.9 ± 11.0	83.5 ± 12.1	78.9 ± 12.1^*^	79.8 ± 12.2^△^
FBG [mmol/L (mg/dl)]	5.67 ± 1.12 (102.2 ± 20.2)	5.35 ± 1.21 (96.4 ± 21.8) ^*^	5.39 ± 1.21 (97.1 ± 21.8)	5.24 ± 1.16 (94.4 ± 20.9)	5.23 ± 1.41 (94.2 ± 25.4)	5.23 ± 1.36 (94.2 ± 24.5)^△^
TG [mmol/L (mg/dl)]	2.53 ± 1.93 (224 ± 171)	1.51 ± 1.31 (134 ± 116) ^*^	1.62 ± 1.43 (143 ± 127)	2.39 ± 2.03 (212 ± 180)	1.60 ± 1.43 (142 ± 126) ^*^	1.75 ± 1.59 (155 ± 141) ^△^
TC [mmol/L (mg/dl)]	5.00 ± 0.99 (193 ± 38.3)	4.52 ± 0.85 (175 ± 32.9) ^*^	4.58 ± 0.88 (177 ± 34.0)	4.71 ± 0.86 (161 ± 33.2)	4.49 ± 0.85 (174 ± 32.9) ^*^	4.53 ± 0.85 (175 ± 32.9) ^△^
HDL [mmol/L (mg/dl)]	1.11 ± 0.25 (42.9 ± 9.7)	1.25 ± 0.32 (48.3 ± 12.4) ^*^	1.24 ± 0.32 (48.0 ± 12.4)	1.10 ± 0.29 (42.5 ± 11.2)	1.31 ± 0.37 (50.7 ± 14.3) ^*^	1.27 ± 0.36 (49.1 ± 13.9) ^△^
LDL [mmol/L (mg/dl)]	2.71 ± 0.72 (105 ± 27.8)	2.45 ± 0.65 (94.8 ± 25.1) ^*^	2.48 ± 0.66 (95.9 ± 25.5)	2.70 ± 0.73 (104 ± 28.2)	2.50 ± 0.71 (96.7 ± 27.5) ^*^	2.53 ± 0.71 (97.8 ± 27.5)^△^
BUN [mmol/L (mg/dl)]	5.89 ± 1.47 (16.5 ± 4.1)	5.38 ± 1.42 (15.1 ± 4.0) ^*^	5.44 ± 1.43 (15.2 ± 4.0)	5.36 ± 1.43 (15.0 ± 4.0)	5.04 ± 1.28 (14.! ± 3.6) ^*^	5.10 ± 1.29 (14.3 ± 3.6) ^△^
Cr [μmol/L (mg/dl)]	79.3 ± 14.8 (0.90 ± 0.17)	67.8 ± 18.5 (0.77 ± 0.21) ^*^	69.1 ± 18.5 (0.78 ± 0.21)	93.7 ± 25.5 (1.06 ± 28.6)	81.6 ± 18.8 (0.92 ± 0.21) ^*^	83.9 ± 20.8 (0.95 ± 0.24) ^△^
UA [μmol/L (mg/dl)]	457.6 ± 50.1 (7.69 ± 0.84)	297.1 ± 64.6 (4.99 ± 1.09) ^*^	314.5 ± 80.8 (5.29 ± 1.36)	464.6 ± 53.3 (7.81 ± 0.90)	310.6 ± 62.3 (5.22 ± 1.05) ^*^	339.4 ± 85.4 (5.71 ± 1.44) ^△^

*UA* uric acid, *BMI* body mass index, *SBP* systolic blood pressure, *DBP* Diastolic blood pressure, *FBG* fasting blood glucose, *TG* triglycerides, *TC* total cholesterol, *HDL* high density lipoprotein, *LDL* low density lipoprotein, *BUN* blood urea nitrogen, *Cr* creatinine

^*^ *P* < 0.01comparing participants with hyperuricemia to those with non-hyperuricemia within 2009 group or 2019 group

^△^*P* < 0.01 comparing between 2009 and 2019 participants

### The prevalence of HUA in all age groups in the 2009 and 2019 participants

**Fig. 1:**
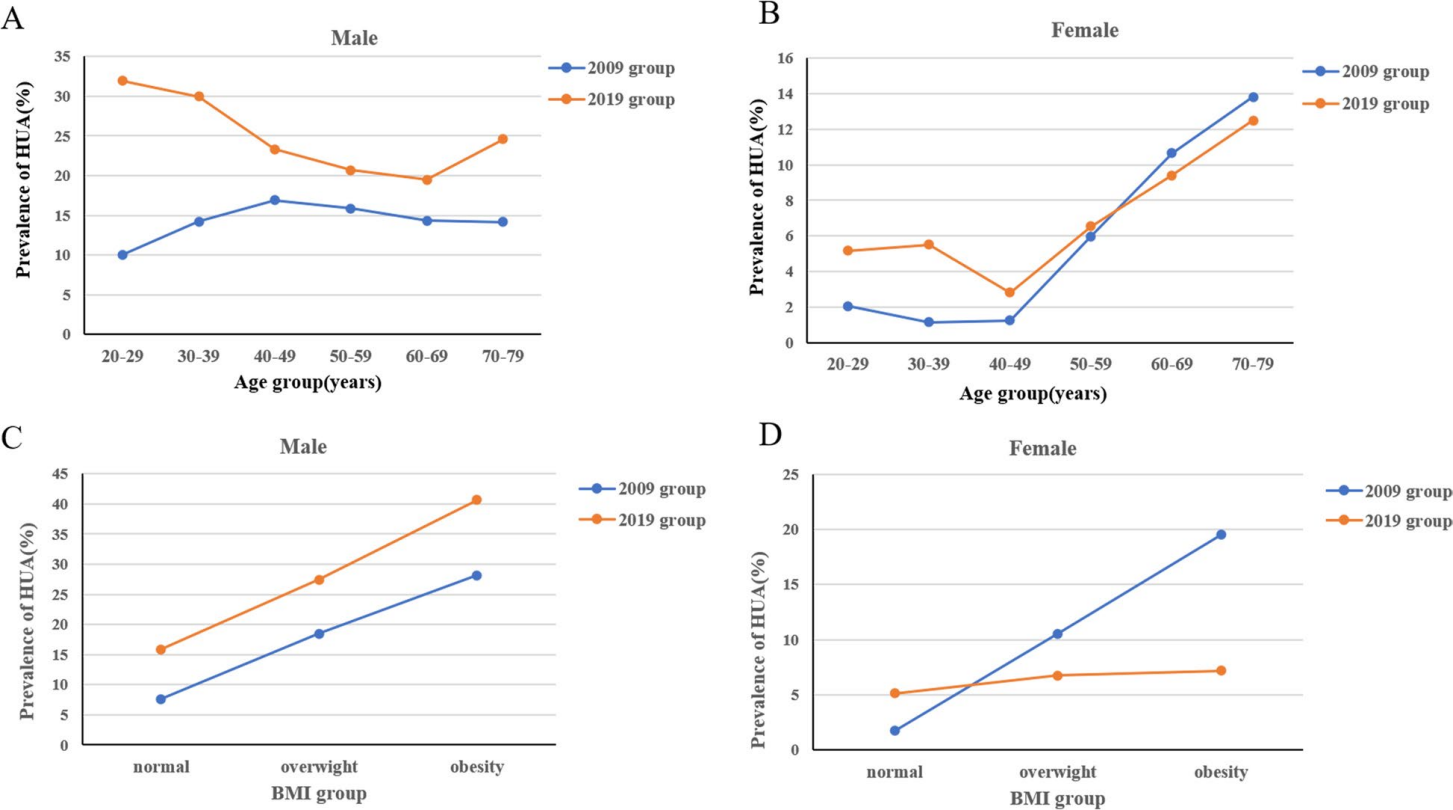
The prevalence of HUA of males and females in different age groups and BMI groups in 2009 and 2019 participants

In Table 2 and Fig. 1, we presented the prevalence of HUA and serum UA levels in all age groups in the 2009 and 2019 participants. In the 2019 group, the overall prevalence of HUA was 18.7% (95% CI 18.0, 19.4), which was significantly higher than that in the 2009 group (11.1% [95% CI 10.2, 12.0] (*p* < 0.01). HUA was more common in males than in females both in the 2009group (15.0% [95% CI 13.7, 16.2] vs. 3.8% [95% CI 2.9, 4.7], *p* < 0.01) and 2019 group (24.8% [95% CI 23.9, 25.7] vs. 5.6% [95% CI 4.8, 6.3] *p* < 0.01).

**Table 2:** Prevalence of HUA and UA levels in 2009 and 2019 participants in each age category

		20–29 years		30–39 years		40–49 years		50–59 years		60–69 years		70–79 years		overall	
		F	M	F	M	F	M	F	M	F	M	F	M	F	M
UA level	2009 participants	245 ± 51.3 (4.12 ± 0.86)	343 ± 61.5 (5.77 ± 1.03) ^△^	235 ± 47.0 (3.95 ± 0.79)	347 ± 66.1 (5.83 ± 1.11) ^△^	240 ± 48.6 (4.03 ± 0.82)	356 ± 72.0 (5.98 ± 1.21) ^△^	261 ± 55.4 (4.39 ± 0.93)	349 ± 71.7 (5.87 ± 1.21) ^△^	281 ± 61.6 (4.72 ± 1.03)	345 ± 73.1 (5.80 ± 1.23)^△^	289 ± 67.8 (4.86 ± 1.14)	346 ± 74.9 (5.82 ± 1.26) ^△^	250 ± 55.2 (4.20 ± 0.93)	349 ± 70.0 (5.87 ± 1.18) ^*△^
[μmol/L (mg/dl)]	2019 participants	270 ± 49.7 (4.54 ± 0.84) ^**^	389 ± 79.6 (6.54 ± 1.34) ^**△^	263 ± 52.5 (4.42 ± 0.88) ^**^	387 ± 73.6 (6.51 ± 1.24) ^**△^	253 ± 50.5 (4.25 ± 0.85) ^**^	373 ± 73.1 (6.27 ± 1.23)^**△^	274 ± 52.6 (4.61 ± 0.89) ^**^	362 ± 75.0 (6.09 ± 1.26)^**△^	284 ± 57.0 (4.77 ± 0.96)	359 ± 75.5 (6.04 ± 1.27)^**△^	291 ± 58.5 (4.89 ± 0.98)	363 ± 84.0 (6.10 ± 1.41) ^**△^	267 ± 53.2 (4.49 ± 0.89)^**^	373 ± 76.2 (6.27 ± 1.28)^**△^
Prevalence of HUA, % (95% CI)	2009 participants	2.0 (0.3–3.8)	10.0 (6.3–13.8)^△^	1.2 (0.1–2.2)	14.2 (11.7–16.7)^△^	1.3 (0.3–2.3)	16.9 (14.5–19.3)^△^	6.0 (2.9–9.0)	15.8 (12.7–18.9)^△^	10.7 (6.3–15.0)	14.3 (10.7–17.9)	13.8 (6.7–20.9)	14.1 (10.2–17.9)	3.8 (2.9–4.7)	15.0 (13.7–16.2)^△^
	2019 participants	5.2 (3.5–6.9) ^**^	31.9 (28.9–34.9)^**△^	5.5 (4.1–6.9) ^**^	29.9 (27.9–32.0)^△^	2.8 (1.8–3.9)	23.4 (21.6–25.3)^**△^	6.5 (4.8–8.3)	20.7 (18.9–22.5)^*△^	9.4 (6.3–12.5)	19.5 (17.0–22.0)^**△^	12.5 (6.7–18.3)	24.7 (20.6–28.7)^**△^	5.6 (4.8–6.3) ^**^	24.8 (23.9–25.7)^**△^

*UA* uric acid, *HUA* hyperuricemia, *F* female, *M* male, *95% CI*, 95% confidence interval

^*^ *P* < 0.05 comparing with 2009 participants

^**^ *P* < 0.01 comparing with 2009 participants

^△^*P* < 0.01 comparing male participants with female

In females, the prevalence of HUA in participants aged 20–29 and 30–39 years in the 2019 was relatively higher than that in the 2009 (*p* < 0.01). There was no difference in the prevalence of HUA in participants aged 40–49, 50–59, 60–69 and 70–79 years between 2009 and 2019 group. In the 2009 and 2019 groups, the prevalence of HUA in elderly females (≥ 60 years old) was significantly higher than that of young women and middle-aged women (20–59 years old), and the prevalence of HUA reached its peak at the age of 70–79.

For males, the prevalence of HUA in all age groups in 2019 participants was significantly higher than that in the 2009 participants (*p* < 0.01). The prevalence of HUA among young men (20–29 and 30–39 years old) was significantly higher than those of the middle-aged and elderly men in the 2019 participants. The prevalence of HUA in males aged 20–29 years in the 2019 participants (31.9% [95% CI 28.9, 34.9]) was nearly triple that of the same age group in the 2009 participants (10.0% [95% CI 6.3, 13.8]), while the prevalence of males aged 30–39 years in the 2019 participants (29.9% [95% CI 27.9, 32.0]) was more than twice that of the same age group in 2009 (14.2% [95% CI 11.7, 16.7]). In the 2019 participants, young males aged 20–29 years had the highest prevalence of HUA, while the HUA prevalence reached its peak in the middle-aged males (aged 40–49 years) in the 2009 participants.

### Serum UA levels in all age groups in the 2009 and 2019 participants

The change trend of serum UA levels of all age categories was similar to the change in the prevalence of HUA in both men and women. Serum UA levels of both males and females in the 2019 group were significantly higher than those in the 2009 group [males: 373 ± 76.2 vs. 349 ± 70.0 μmol/L (6.27 ± 1.28 vs. 5.87 ± 1.18 mg/dL), *p* < 0.01; females: 267 ± 53.2 vs. 250 ± 55.2 μmol/L (4.49 ± 0.89 vs. 4.20 ± 0.93 mg/dL), *p* < 0.01]. The overall UA levels in males were dramatically higher than those in females both in the 2009 and 2019 groups [in 2009: 349 ± 70.0 vs. 250 ± 55.2 μmol/L (5.87 ± 1.18 vs. 4.20 ± 0.93 mg/dL), *p* < 0.01; in 2019: 373 ± 76.2 vs. 267 ± 53.2 μmol/L (6.27 ± 1.28 vs. 4.49 ± 0.89 mg/dL), *p* < 0.01)]. Serum UA levels of all age groups in males were also obviously higher than those in females both in the 2009 participants and 2019 participants. Serum UA levels in young and middle-age female participants (20–29, 30–39, 40–49 and 50–59 years old) in the 2019 were significantly higher than those in the 2009. However, there was no difference in the UA levels of elderly females (60–69 and 70–79 age group) between the 2019 participants and 2009 participants. In males, UA levels of all age groups in the 2019 participants were significantly higher than those in the 2009 participants (Table 2 and Fig. 1).

### The prevalence of HUA in different BMI groups in the 2009 and 2019 participants

All participants were divided into three BMI groups, including normal weight (BMI < 24 kg/m^2^), overweight (24 kg/m^2^ ≤ BMI < 28 kg/m^2^) and obesity (BMI ≥ 28 kg/m^2^). The prevalence of HUA in different BMI groups in the 2009 and 2019 participants were presented in Table 3 and Fig. 1. In males, the prevalence of HUA in all BMI groups in the 2019 participants were significantly higher than those in the 2009 participants. The prevalence of HUA in obese men was markedly higher than that of males with normal weight and overweight both in the 2009 and 2019 participants. We found that the prevalence of HUA in males in both 2009 and 2019 participants was dramatically increased along with the elevated BMI value. However, this change trend was not significant in females.

**Table 3:** Prevalence of HUA in 2009 and 2019 participants in BMI category

		Normal weight (BMI < 24 kg/m^2^)		Overweight (24 kg/m^2^ ≤ BMI < 28 kg/m^2^)		Obese (BMI ≥ 28 kg/m^2^)	
		F	M	F	M	F	M
Prevalence of HUA, % (95% CI)	2009 participants	1.7 (1.0–2.4)	8.1 (6.7–9.6)^△^	10.5 (7.1–14.0) ^$^	18.5 (16.5–20.5)^△$^	19.5 (6.8–32.2) ^$^	28.1 (23.2–33.0)^△$&^
	2019 participants	5.2 (4.4–6.0) ^**^	15.8 (14.6–17.1)^**△^	6.8 (5.0–8.5) ^*^	27.4 (26.0–28.8) ^**△$^	7.2 (3.1–11.3) ^*^	40.7 (37.9–43.4) ^**△$&^

^*^ *p* < 0.05 comparing with 2009 participants

^**^ *p* < 0.01 comparing with 2009 participants

^△^ *p* < 0.01 comparing male participants with females

^$^ *p* < 0.01 comparing with participants with normal weight

^&^ *p* < 0.01 comparing with participants with overweight, *BMI* body mass index, *HUA* hyperuricemia, *95% CI*, 95% confidence interval

The prevalence of HUA in females with normal weight in the 2019 participants was significantly higher than that in the 2009 participants (5.2% [95% CI 4.4, 6.0]), vs. 1.7% [95% CI 1.0, 2.4]), *p* < 0.01). However, the prevalence of HUA in females with obesity and overweight was obviously lower in the 2019 participants compared with that in the 2009 participants (6.8% [95% CI 5.0, 8.5]) vs. 10.5% [95% CI 7.1, 14.0]) in obese group, *p* < 0.05; 7.2% [95% CI 3.1, 11.3]) vs. 19.5% [95% CI 6.8, 32.2]) in overweight group, *p* < 0.05). In addition, the prevalence of HUA of obese and overweight females in the 2009group were higher than that of females with normal weight. There was no significant difference in the prevalence of HUA between obese and overweight females in the 2009 participants. In the 2019 group, there was no significant difference in the prevalence of HUA of females among three BMI groups.

### Risk factors for HUA

The relationship of biochemical parameters and HUA using spearman’s correlation analysis was shown in Table 4. BMI, BH, BW, FBG, TG, CHOL, LDL, Cr and BUN were positively associated with serum UA levels and HUA in the overall participants in both 2009 and 2019, while HDL was negatively related to UA levels and HUA. Moreover, there were significant sex differences in both UA levels and HUA prevalence in the two groups of 2009 and 2019. Men had higher levels of UA and were more likely to develop HUA compared to women. In the 2009 group, age was positively correlated with serum UA levels and HUA, whereas in the 2019 group, age was negatively associated with HUA, and was not correlated with serum UA levels.

**Table 4:** Correlations of UA levels and HUA with related variables in 2009 and 2019 participants

		Age	BMI	BH	BW	sex	FBG	TG	TC	HDL	LDL	Cr	BUN
UA level	2009 participants	0.134 (*p* < 0.01)	0.458 (*p* < 0.01)	0.482 (*p* < 0.01)	0.570 (*p* < 0.01)	-0.617 (*p* < 0.01)	0.191 (*p* < 0.01)	0.458 (*p* < 0.01)	0.187 (*p* < 0.01)	-0.333 (*p* < 0.01)	0.196 (*p* < 0.01)	0.239 (*p* < 0.01)	0.596 (*p* < 0.01)
	2019 participants	0.003 (*p* = 0.756)	0.396 (*p* < 0.01)	0.474 (*p* < 0.01)	0.562 (*p* < 0.01)	-0.610 (*p* < 0.01)	0.099 (*p* < 0.01)	0.416 (*p* < 0.01)	0.131 (*p* < 0.01)	-0.426 (*p* < 0.01)	0.177 (*p* < 0.01)	0.585 (*p* < 0.01)	0.197 (*p* < 0.01)
HUA	2009 participants	0.074 (*p* < 0.01)	0.242 (*p* < 0.01)	0.125 (*p* < 0.01)	0.237 (*p* < 0.01)	-0.169 (*p* < 0.01)	0.146 (*p* < 0.01)	0.263 (*p* < 0.01)	0.153 (*p* < 0.01)	-0.152 (*p* < 0.01)	0.119 (*p* < 0.01)	0.112 (*p* < 0.01)	0.235 (*p* < 0.01)
	2019 participants	-0.034 (*p* < 0.01)	0.242 (*p* < 0.01)	0.193 (*p* < 0.01)	0.292 (*p* < 0.01)	-0.229 (*p* < 0.01)	0.066 (*p* < 0.01)	0.269 (*p* < 0.01)	0.104 (*p* < 0.01)	-0.237 (*p* < 0.01)	0.101 (*p* < 0.01)	0.285 (*p* < 0.01)	0-.092 (*p* < 0.01)

*UA* uric acid, *BMI* body mass index, *BH* body height, *BW* body weight, *FBG* fasting blood glucose, *TG* triglycerides, *TC* total cholesterol, *HDL* high density lipoprotein, *LDL* low density lipoprotein, *Cr* creatinine, *BUN* blood urea nitrogen

**Fig. 2:**
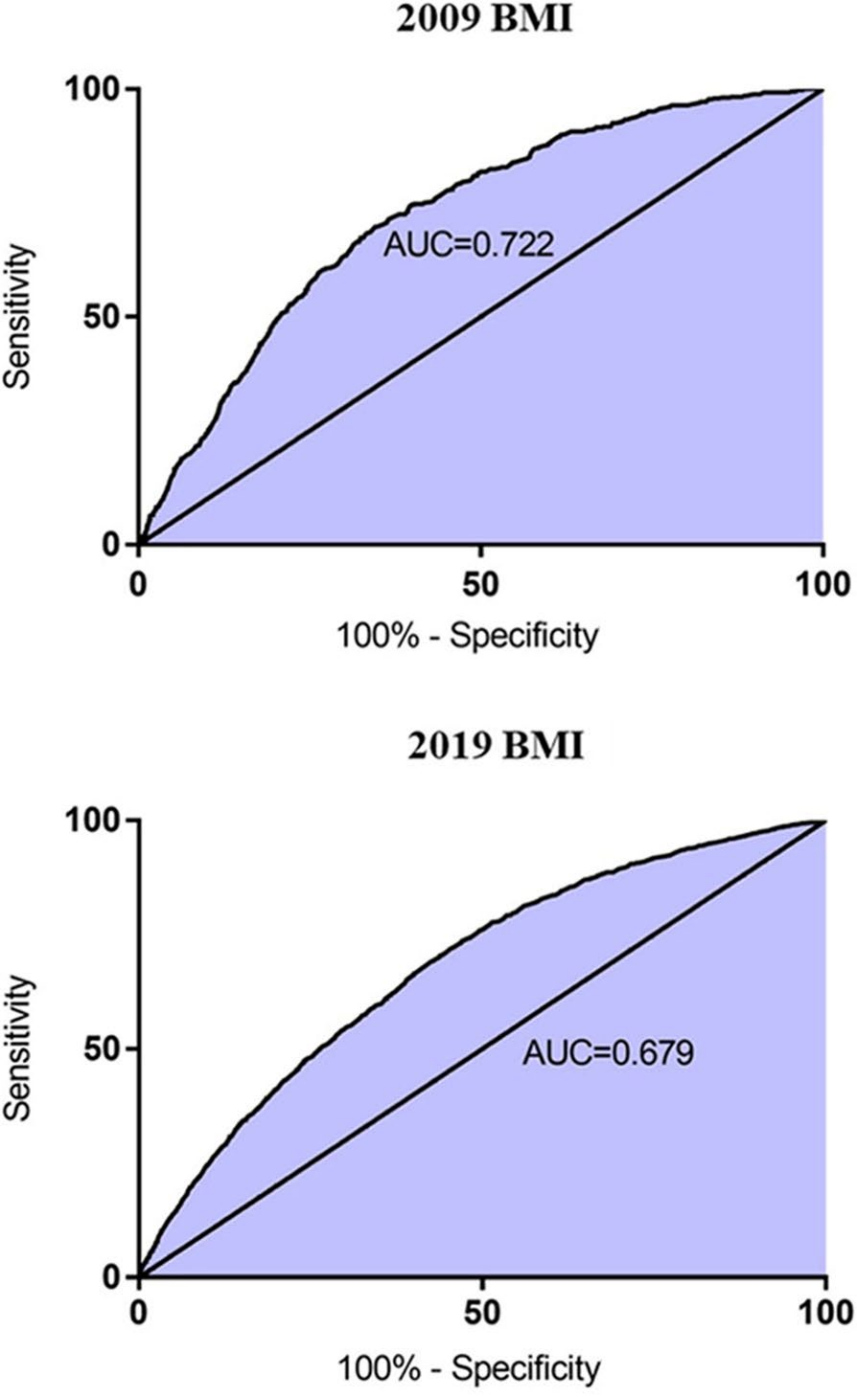
Receiver-operating characteristic (ROC) curves for the ability of BMI to discriminate the HUA patients from the non-HUA participants in 2009 participants and 2019 participants. The area under the ROC curve (AUC) = 0.722 for BMI in 2009 participants, *P* < 0.001, 95%CI 0.701-0.743; AUC = 0.679 for BMI in 2019 participants, *P* < 0.001, 95%CI 0.667-0.691.

To further investigate the associations between HUA and BMI, unadjusted and multivariate adjusted logistic regression analyses were performed. BMI was significantly positively associated with HUA in the 2009 participants (OR: 1.212, 95% confidence interval (CI): 1.133–1.296, *p* < 0.01) and 2019 participants (OR: 1.200, 95% CI: 1.161–1.241, *p* < 0.01). Those associations also persisted after adjustment for age, sex, BUN, Cr, FBG, TG, CHOL, HDL and LDL (2009 group: OR: 1.136, 95% CI: 1.054–1.224, *p* < 0.01; 2019 group: OR: 1.102, 95% CI: 1.063–1.43, *p* < 0.01). ROC curves were conducted to determine the cut-off, sensitivity and specificity of the risk factors which could potentially predict HUA in the 2009 and 2019 groups. The area under curves (AUC) of BMI were presented in Fig. 2. As demonstrated by the ROC curves, BMI > 24.48 kg/m^2^ displayed good capacities to discriminate HUA from non-HUA in the 2009 participants (AUC = 0.722, *p* < 0.01, 95% CI 0.628–0.849), while BMI > 23.84 kg/m^2^ could predict HUA in the 2019 participants (AUC = 0.679, *p* < 0.01, 95%CI 0.667–0.691).

## Discussion

Our research is the first study on the changes in prevalence of HUA in Eastern China in recent decade. We found no statistically significant differences in age distribution, sex ratio, weight, BMI, and other major socio-demographic characteristics between the two medical examination populations we studied and other large-scale medical examination populations in our province, indicating that the populations we studied were well represented [28, 29]. In this survey, the prevalence of HUA in the 2009 participants and 2019 participants were 11.1% and 18.7%, respectively. The overall prevalence of HUA in the 2019 group was significantly higher than that in the 2009 group both in males and females. Liu et al. [30] have demonstrated that the prevalence of HUA in the Chinese population has increased rapidly from 2000 to 2014. In the United States, the prevalence of HUA has increased significantly from 18.2% in NHANES 1988–1994 to 21.4% in NHANES 2007–2008 [31]. It was reported that the prevalence of HUA among men in the United States (21.2%) was very close to that of women (21.6%) [31]. This is very different from our findings. We found that the prevalence of HUA among men in the Chinese population was significantly higher than that in women.

In addition, our data indicated the prevalence rates of HUA in both males and females in 2019 were significantly higher than those in 2009 (males: 24.8% vs. 15.0%; females: 5.6% vs. 3.8%). Previous studies have reported similar results in other regions of China [32] and in other countries such as Japan and Bangladesh [33, 34]. There was obvious sex difference in the prevalence of HUA in the 2009 and 2019 participants, and the prevalence of HUA in men was much higher than that in women. The reason may be related to differences in estrogen levels, eating habits and weight gain between men and women. Women, influenced by traditional Chinese culture, tend to take on more housework and may also deliberately control weight to maintain their figures. Men with extensive social networks usually accept a high-fat diets or alcohol. Although the prevalence rates of HUA in females aged 20–39 years old were significantly higher in the 2019 group than 2009 group, there was no significant difference of HUA prevalence in females aged 40–79 years old between 2009 and 2019. The prevalence of HUA in females was gradually increased with age starting from the age of 40 both in 2009 and 2019.

HUA is generally considered to be a common disease in middle-aged men, and males aged 40–49 years old was indeed the main population with HUA in 2009. However, in 2019, the prevalence of HUA in young men was higher than that of middle-aged males. Among them, the prevalence of HUA in young men aged 20–29 years old in the 2019 participants (31.9%) was nearly three times of that in males of the same age group in the 2009 participants (10.0%). Therefore, we suggest that young men are the group with the fastest increase in the prevalence of HUA in recent 10 years. In 2019, young men have replaced middle-aged men as the main population with HUA. This condition may be due to unhealthy lifestyles in young men, including high-fat diet and lack of physical exercise, which are considered to be an important determinant of elevated serum UA levels. The high prevalence of HUA not only induces a high risk of gout [35], but is also associated with increased risks of hypertension, diabetes, CVD and CKD [36–38]. The rising prevalence of HUA among young men suggests that the prevalence of gout and other related metabolic disorders is also increasing. Therefore, special attention should be paid to the prevention and treatment of HUA in young males.

In our study, we also found that BMI was significantly associated with HUA in both groups of 2009 and 2019. Obesity has become a global problem and is recognized as a risk factor leading to multiple adverse health consequences [10]. Several studies have demonstrated the relationship between obesity and HUA [34, 39]. A positive correlation between serum UA level and obesity was found in Bangladeshi adults [34]. A Japanese study reported that HUA was significantly associated with central obesity in obese populations rather than in normal-weight men and women [33]. In the Chinese population, obesity was found to be a crucial risk factor for HUA in females [39]. Therefore, our results were consistent with the previous findings. In addition, we found that BMI > 24.48 kg/m^2^ and BMI > 23.84 kg/m^2^ could display good capacities to discriminate patients with HUA from non-HUA in the 2009 participants and 2019 participants, respectively. This result further confirmed the association between HUA and obesity. Nevertheless, we should notice that the cut-off of BMI used to predict the HUA has begun to decline in the past decade. This may imply that the people with a lower BMI than before are also likely to suffer from HUA, but the reason for this change is still unclear. It may be related to changes in diet and lifestyles to a certain extent. For example, a recent study showed that the Chinese diet was shifting to high-fat and high-energy-dense foods [40]. The consumption of vegetable oils, animal-derived foods and processed foods that are rich in refined starch, sugar, salt and unhealthy fats continued to increase. The consumption of staple foods has gradually changed from the traditional staple foods of coarse grains to refined cereals [41]. According to the Global Burden of Diseases database, the burden of disease in urban areas of China, which are associated with individual behaviors and habits such as unhealthy diet, drinking, smoking and lack of physical exercise, is steadily increasing [42]. Moreover, previous studies have reported that increased consumption of tea and high-fructose corn syrup could also influence the level of UA [43, 44]. For green tea, a study among Chinese in Singapore has revealed that daily green tea drinkers exhibited a twofold increase in association with HUA compared with nondrinkers [45]. The relationship between black tea and UA was contradictory due to differences in baseline UA levels between studies [43]. It has been reported that high-fructose corn syrup can increase blood UA levels. Previous studies have shown that the metabolism of fructose stimulates UA production, and long-term fructose administration inhibits renal excretion of UA, which in turn leads to increased serum UA levels [44].

Our research also has several limitations. Firstly, all subjects were from a single center, which may limit the extrapolation of our conclusions. Secondly, most of the elderly who participated in this study were in good health, and patients with serious illnesses were unable to participate in our study, which may lead to an underestimation of the prevalence of HUA among the elderly. Thirdly, due to the limited information in our physical examination database, more relevant factors of HUA, such as educational status, drinking history, smoking history, medication and past medical history (eg, gout and HUA comorbidities) were not able to be assessed.

## Conclusions

The prevalence rate of HUA in Eastern China has been increasing rapidly in the past 10 years. Young men aged 20–29 years in the 2019 group replaced the middle-aged males (40–49 years old) in the 2009 group, and became the main population of male HUA in the 2019group. BMI was positively correlated with HUA, and BMI displayed a good capacity to discriminate the patients with HUA from non-HUA in both the 2009 participants and 2019 participants.

## Data Availability

The datasets used and analysed during the current study are available from the corresponding author on reasonable request.

## Abbreviations

HUA: Hyperuricemia
UA: Uric acid
BMI: Body mass index
BH: Body height
BW: Body weight
SBP: Systolic blood pressure
DBP: Diastolic blood pressure
FBG: Fasting blood-glucose
TG: Triglyceride
CHOL: Cholesterol
HDL: High-density lipoprotein
LDL: Low-density lipoprotein
Cr: Creatinine
BUN: Blood urea nitrogen
CI: Confidence interval
CVD: Cardiovascular disease
CKD: Chronic kidney disease
SPSS: Statistical package for social sciences
ROC: Receiver-operating characteristic
AUC: Area under curves

## References

[1] Ichida K, Matsuo H, Takada T, Nakayama A, Murakami K, Shimizu T (2012). Decreased extra-renal urate excretion is a common cause of hyperuricemia. Nat Commun.

[2] Junxia Su, Wei Y, Liu M, Liu T, Li J, Ji Y (2014). Anti-hyperuricemic and nephroprotective effects of Rhizoma Dioscoreae septemlobae extracts and its main component dioscin via regulation of mOAT1, mURAT1 and mOCT2 in hypertensive mice. Arch Pharm Res.

[3] Dong X, Zhang H, Wang F, Liu X, Yang K, Runqi Tu (2019). Epidemiology and prevalence of hyperuricemia among men and women in Chinese rural population: The Henan Rural Cohort Study. Mod Rheumatol.

[4] Georgiana Cabău, Tania O Crișan, Viola Klück, Radu A Popp, Leo A B Joosten. Urate-induced immune programming: Consequences for gouty arthritis and hyperuricemia. Immunol Rev. 2020; 294(1):92–105. 10.1111/imr.12833. 10.1111/imr.12833 PMC7065123 31853991

[5] Ravi K Narang, Greg G Gamble, Ruth Topless, Murray Cadzow, Lisa K Stamp, Tony R Merriman, et al. Assessing the Relationship Between Serum Urate and Urolithiasis Using Mendelian Randomization: An Analysis of the UK Biobank. Am J Kidney Dis. 2021; 78 (20): 210–218. 10.1053/j.ajkd.2020.11.018. 10.1053/j.ajkd.2020.11.018 33400963

[6] Richard J Johnson, Takahiko Nakagawa, Diana Jalal, Laura Gabriela Sánchez-Lozada, Duk-Hee Kang, Eberhard Ritz. Uric acid and chronic kidney disease: which is chasing which? Nephrol Dial Transplant. 2013; 28 (9): 2221–2228. 10.1093/ndt/gft029. 10.1093/ndt/gft029 PMC4318947 23543594

[7] Abbas Dehghan, Mandy van Hoek, Eric J G Sijbrands, Albert Hofman, Jacqueline C M Witteman. High serum uric acid as a novel risk factor for type 2 diabetes. Diabetes care. 2008; 31(2):361–362. 10.2337/dc07-1276. 10.2337/dc07-1276 17977935

[8] Peter C Grayson, Seo Young Kim, Michael LaValley, Hyon K Choi. Hyperuricemia and incident hypertension: a systematic review and meta-analysis. Arthritis Care Res (Hoboken). 2011; 63(1):102–110. 10.1002/acr.20344 PMC3016454 20824805

[9] Seo Young Kim, James P Guevara, Kyoung Mi Kim, Hyon K Choi, Daniel F Heitjan, Daniel A Albert. Hyperuricemia and coronary heart disease: a systematic review and meta-analysis. Arthritis Care Res (Hoboken). 2010; 62(2):170–180. 10.1002/acr.20065. 10.1002/acr.20065 PMC3156692 20191515

[10] Zhang L, Wang F, Wang Li, Wang W, Liu B, Liu J (2012). Prevalence of chronic kidney disease in China: a cross-sectional survey. Lancet.

[11] Qi D, Liu J, Wang C, Wang L, Zhang X, Lin Q (2020). Sex-specific differences in the prevalence of and risk factors for hyperuricemia among a low-income population in China: a cross-sectional study. Postgrad Med.

[12] Zifeng Liu, Xiaoting Su, Mianli Xiao, Peien Zhou, Jianwei Guo, Yixiang Huang, et al. Association between Eating Away from Home and Hyperuricemia: A Population-Based Nationwide Cross-Sectional Study in China. Biomed Res Int. 2019; 2019: 2792681. eCollection. 10.1155/2019/2792681. 10.1155/2019/2792681 PMC6794973 31687384

[13] Liu H, Zhang X-M, Wang Y-L, Liu B-C (2014). Prevalence of hyperuricemia among Chinese adults: a national cross-sectional survey using multistage, stratified sampling. J Nephrol.

[14] Wang R, Tang Z, Sun F, Diao LJ (2018). Prevalence of hyperuricemia in the elderly in 7 areas of China. Zhonghua Liu Xing Bing Xue Za Zhi.

[15] Zheng ML, Lai YH, He XN, Tan XW  (2008). Correlation of detection of hyperuricemia with hypertension in healthy population in Guangzhou City. China Trop Med.

[16] Han XH, Yao XY, Fang XS (2008). The prevalence of hyperuricemia and abnormal of lipid and glucose in medical examination population. Shanxi Med J.

[17] Cao YJ, Liu Y, Li T, Yan BS, Wang ZZ, Yang XL (2010). Investigation of the increasing of policemen in blood uric acid and triglyceride in Haikou. Hainan Med J.

[18] Gao CJ, Jiang YG, Tang ZL (2008). Epidemiological analysis of hyperuricemia in 39824 healthy subjects in Anhui province. J Pract Med.

[19] Zhang JM, Yao LN, Li H (2009). Study on hyperuricemia in the physical examination population in Zhengzhou area. J Zhengzhou University.

[20] Zhang Q, Gong H, Lin C, Liu Q, Baima Y, Wang Y (2020). The prevalence of gout and hyperuricemia in middle-aged and elderly people in Tibet Autonomous Region, China: A preliminary study. Medicine (Baltimore).

[21] Chen M-Y, Zhao C-C, Li T-T, Zhu Y, Tian-Pei Yu, Bao Y-Q (2017). Serum uric acid levels are associated with obesity but not cardio-cerebrovascular events in Chinese inpatients with type 2 diabetes. Sci Rep.

[22] Zhu C, Cui R, Gao M, Rampersad S, You H, Sheng C (2017). The Associations of Serum Uric Acid with Obesity-Related Acanthosis nigricans and Related Metabolic Indices. Int J Endocrinol.

[23] Duan Y, Liang W, Zhu L, Zhang T, Wang L, Nie Z (2015). Association between serum uric acid levels and obesity among university students (China). Nutr Hosp.

[24] Yang C, Yang S, Feng C, Zhang C, Weiwei Xu, Zhang L (2018). Associations of hyperuricemia and obesity with remission of nonalcoholic fatty liver disease among Chinese men: A retrospective cohort study. PLoS ONE.

[25] Zhou H, Ma ZF, Lu Y, Du Y, Shao J, Wang L, Wu Q, Pan B, Zhu W, Zhao Q, Wei H. Elevated serum uric acid, hyperuricaemia and dietary patterns among adolescents in mainland China. 2020, 8;33(4): 487–493. 10.1515/jpem-2019-0265. 10.1515/jpem-2019-0265 32069235

[26] G Neil Thomas, Sai-Yin Ho, Edward D Janus, Karen S L Lam, Anthony J Hedley, Tai Hing Lam. Hong Kong Cardiovascular Risk Factor Prevalence Study Steering Committee, The US National Cholesterol Education Programme Adult Treatment Panel III (NCEP ATP III) prevalence of the metabolic syndrome in a Chinese population. Diabetes Res Clin Pract. 2005; 67(3): 251–257. 10.1016/j.diabres.2004.07.022. 10.1016/j.diabres.2004.07.022 15713358

[27] Zhou B-F (2005). Predictive values of body mass index and waist circumference for risk factors of certain related diseases in Chinese adults: study on optimal cut-off points of body mass index and waist circumference in Chinese adults. Biomed Environ Sci.

[28] Lu Yun, Qi Hua-jin, Li Feng, Fang Ning-yuan, Wang Ling, Zou Hai-hong, Fan Yu-peng, Shen Zhen-hai. The investigation of ideal cardiovascular health behaviors and factors among young and middle-aged populations in Suzhou, Wuxi and Changzhou cities of Jiangsu province. Chin J Hypertens, 2015, 23 (10): 964–973. 10.16439/j.cnki.1673-7245.2015.10.022.

[29] Sun Jie, Zhou Weihong, Gu Tianwei, Wang Jing, Zhu Dalong, Bi Ye. Prevalence of overweight and obesity and its relationship with the risk factors of cardiovascular diseases among population for physical examination in Nanjing Drum Tower Hospital. Chin J Endocrinol Metab, 37(1): 39–44. 10.3760/cma.j.cn311282-20200430-00318.

[30] Liu R, Han C, Di Wu, Xia X, Jianqiu Gu, Guan H (2015). Prevalence of Hyperuricemia and Gout in Mainland China from 2000 to 2014: A Systematic Review and Meta-Analysis. BioMed Res Int.

[31] Katrine L Wallace, Aylin A Riedel, Nancy Joseph-Ridge, Robert Wortmann. Increasing prevalence of gout and hyperuricemia over 10 years among older adults in a managed care population. J Rheumatol.2004; 31(8): 1582–1527. PMID: 15290739. 15290739

[32] Wang Q, Wang C, Xue J, Chen MM, Sun HW, Jiang M (2021). Characteristics of serum uric acid distribution in occupation, age, gender groups and its influencing factors in physical examination subjects in Nanjing from 2012 to 2016. Zhonghua Nei Ke Za Zhi.

[33] Shirasawa T, Ochiai H, Yoshimoto T, Nagahama S, Watanabe A, Yoshida R (2020). Cross-sectional study of associations between normal body weight with central obesity and hyperuricemia in Japan. BMC Endocr Disord.

[34] Ali N, Perveen R, Rahman S, Mahmood S, Rahman S, Islam S (2018). Prevalence of hyperuricemia and the relationship between serum uric acid and obesity: A study on Bangladeshi adults. PLoS ONE.

[35] Somchai Uaratanawong, S Suraamornkul, S Angkeaw, R Uaratanawong. Prevalence of hyperuricemia in Bangkok population. Clin Rheumatol. 2011; 30 (7): 887–893. 10.1007/s10067-011-1699-0. 10.1007/s10067-011-1699-0 21302126

[36] Vitool Lohsoonthorn, Bodi Dhanamun, Michelle A Williams. Prevalence of hyperuricemia and its relationship with metabolic syndrome in Thai adults receiving annual health exams. Arch Med Res. 2016; 37(7):883–889. 10.1016/j.arcmed.2006.03.008. 10.1016/j.arcmed.2006.03.008 16971230

[37] Ismail Sari, Servet Akar, Betul Pakoz, Ali Riza Sisman, Oguz Gurler, Merih Birlik, et al. Hyperuricemia and its related factors in an urban population, Izmir, Turkey. Rheumatol Int. 2009; 29(8):869–874. 10.1007/s00296-008-0806-2. 10.1007/s00296-008-0806-2 19048257

[38] Yanyan Zhu, Bhavik J Pandya, Hyon K Choi. Prevalence of gout and hyperuricemia in the US general population: the National Health and Nutrition Examination Survey 2007–2008. Arthritis Rheum. 2011; 63(10):3136–3141. 10.1002/art.30520. 10.1002/art.30520 21800283

[39] Song P, Wang He, Xia W, Chang X, Wang M, An L (2018). Prevalence and correlates of hyperuricemia in the middle-aged and older adults in China. Sci Rep.

[40] Xiao Chang, Ruth S DeFries, Liming Liu, Kyle Davis. Understanding dietary and staple food transitions in China from multiple scales. Plos One. 2018; 13(4): e0195775. 10.1371/journal.pone.0195775. 10.1371/journal.pone.0195775 PMC5915834 29689066

[41] Han A, Sun T, Ming J, Chai Li, Liao X (2020). Are the Chinese Moving toward a Healthy Diet? Evidence from Macro Data from 1961 to 2017. Int J Environ Res Public Health.

[42] Li X, Song J, Lin T, Dixon J, Zhang G, Ye H (2016). Urbanization and health in China, thinking at the national, local and individual levels. Environ Health.

[43] Towiwat P, Li ZG. The association of vitamin C, alcohol, coffee, tea, milk and yogurt with uric acid and gout. Int J Rheum Dis 2015;18(5). 10.3390/nu9040395. 10.1111/1756-185X.12622 26082349

[44] Caliceti C, Calabria D, Roda A, Cicero AFG. Fructose Intake, Serum Uric Acid, and Cardiometabolic Disorders: A Critical Review. Nutrients 2017, 18;9(4). 10.3390/nu9040395. 10.3390/nu9040395 PMC5409734 28420204

[45] Teng GG, Tan CS, Santosa A, Saag KG, Yuan JM, Koh WP (2013). Serum urate levels and consumption of common beverages and alcohol among Chinese in Singapore. Arthritis Care Res (Hoboken).

